# Robotic Assisted Laparoscopic Prostatectomy after High Intensity Focused Ultrasound Failure

**DOI:** 10.1155/2017/5980697

**Published:** 2017-01-24

**Authors:** Leon Telis, Seyed Behzad Jazayeri, David B. Samadi

**Affiliations:** Department of Urology, Lenox Hill Hospital, Hofstra Northwell School of Medicine, New York, NY, USA

## Abstract

*Background*. Prostate cancer is the most common cancer diagnosed in men. As new focal therapies become more popular in treatment of prostate cancer, failure cases requiring salvage therapy with either surgical or other techniques are being reported.* Objective*. To report the options in treatment of prostate cancer after recurrence or failure of the primary treatment modality.* Methods*. We report a salvage robotic assisted laparoscopic radical prostatectomy (RALP) for prostate cancer recurrence following high intensity focused ultrasound treatment (HIFU) in the United States.* Results*. A 67-year-old man who underwent HIFU treatment for prostate adenocarcinoma 2 years prior was presented with a rising prostate specific antigen of 6.1 ng/mL to our clinic. A biopsy proven recurrent disease in the area of previous treatment documented the failure of treatment. The patient elected to undergo a salvage RALP. The operation time was 159 minutes. The patient was discharged from the hospital on postoperative day 1 with no complications. The catheter was removed on post-op day 10. The patient reserved sexual function and urinary continence. The PSA levels on 6 months' follow-up are undetectable.* Conclusions*. Salvage RALP is an effective and safe treatment choice for recurrent prostate adenocarcinoma following failed HIFU treatment if operated by an experienced surgeon.

## 1. Introduction

Prostate cancer is the most common cancer among men worldwide. Most of patients are detected at an early stage with a 5-year survival of 98.9% based on 2005–2011 data [1]. The role of active surveillance in prostate cancer management is becoming more prominent in recent years and focal treatments [2] such as high intensity focused ultrasound (HIFU) are becoming more available. HIFU is a new technology based on the physics of ultrasound waves [3, 4]. The technology works on the basis of delivering focused ultrasounds inducing thermal and physical injury to a specific tissue at specific depth in the body [4]. More than 50,000 of men with prostate cancer treated with HIFU are reported in the literature [3]. However, HIFU was approved for the first time in the US in October 2015 after the advisory panel rejected the use of HIFU in treatment of prostate cancer twice in 2014. Here we are reporting a case of HIFU failure treated with robotic assisted laparoscopic prostatectomy (RALP).

## 2. Methods

A 67-year-old man with a past medical history of hypertension, hyperlipidemia, hypothyroidism, and prostate cancer was presented to the urology clinic at Lenox Hill Hospital. In his prior workups in 2012, a transrectal ultrasound-guided biopsy of the prostate confirmed the diagnosis of prostate carcinoma. Biopsy evaluation revealed a Gleason of 7 (3 + 4). The patient underwent focal HIFU treatment at that time, after proper diagnostic procedures. The PSA level remained relatively high at 4.6 ng/mL after HIFU.

Two years later, the patient presented to the urology clinic of Lenox Hill Hospital with a rising PSA level of 6.1 ng/mL detected in his follow-ups.

An informed consent of the patient was obtained to use his clinical information for the present paper.

## 3. Results

At the time of presentation, digital rectal exam revealed the prostate to be benign in consistency aside from the scar from HIFU treatment. A transperineal biopsy was planned, which demonstrated recurrent tumor in the area of previous HIFU therapy at right lateral and the right posterolateral base of prostate with Gleason score of 7 (3 + 4).

On magnetic resonance imaging a 38-gram prostate gland with an intact capsule and no lymphadenopathy in the pelvis was noted. On the preoperative evaluation, the patient had an American Urologic Association score of 5, was urinary continent with no use of pads, and had a sexual health inventory for men score of 24 with use of phosphodiesterase-5 inhibitors.

After discussion of various options of salvage therapy including another focal therapy such as electroporation and another course of HIFU, surgical prostatectomy as salvage therapy was planned for the patient. Salvage RALP was performed using the da Vinci® (Intuitive Surgical, CA, USA) robotic surgical system.

As a reported complication of HIFU therapy is formation of rectal adhesions and fistula formation, during the surgery, posterior dissection of the vas deferens and seminal vesicles was performed first to ensure the prostate was not adherent to the rectal wall prior to any irreversible steps (urethral cut) being taken. A posterior prostatorectal plane was developed bluntly toward the apex of the prostate. Endopelvic fascia was not incised as part of the SMART technique (Samadi Modified Advanced Robotic Technique) in order to decrease risk of injury to neurovascular bundles on the lateral portion of the prostate and preserve erectile function in the patient. Bilateral neurovascular bundles were then spread without any difficulties.

The prostatectomy was followed by bilateral pelvic lymph node dissection. There were no complications during the operation or recovery after general anesthesia. The patient was discharged home on the first day after surgery and urinary catheter was removed on day 10 postoperatively. Surgical pathology report demonstrated prostatic adenocarcinoma, Gleason score of 7 (3 + 4). The tumor measured 1.1 cm involving both right and left lobes of the prostate (pT2cpN0). The patient has reserved his sexual function and urinary continence with current PSA levels <0.01 mg/mL at 6 months' follow-up.

## 4. Discussion

With recent increases in active surveillance as a management option for low-risk prostate cancer [5], patients and clinicians have sought treatment options that produce minimal morbidity in regard to erectile function and urinary continence with maximum oncologic outcomes. More than 50,000 men have been treated for prostate cancer using HIFU technology for men who are seeking definitive treatment of their prostate cancer with minimal possible morbidities. Though HIFU can help to reduce the impact of surgical complications after surgery, a positive biopsy cancer recurrence is seen in 1 out of 5 patients treated primarily with HIFU [6] and retreatment is necessary in up to a third of patients [7].

Lawrentschuk et al. [8] reported that, in 15 men who underwent radical prostatectomy for recurrent prostate carcinoma following failed HIFU treatment, the procedure is feasible but comes with a higher morbidity than for primary surgery. The formation of rectourethral fistula/fibrosis can occur in 1% [9] of patients undergoing primary HIFU therapy causing compromised blood supply and poor tissue quality [8]. Rectal injury rate can therefore potentially be higher for salvage radical prostatectomy. Previously, Murphy et al. [10] reported a case of recurrent prostate cancer following failed HIFU therapy treated with RALP. In the case of Murphy et al. [10], the patient was impotent prior to surgery and gained continence in the course of recovery; however, erectile dysfunction was not responsive to medical therapy. In the current case, erections and continence were both maintained.

Long term oncologic outcome of HIFU treatment in comparison with RALP in localized prostate cancer is not reported in the literature, yet. Proper patient selection is the key to achieve adequate oncologic control with focal therapies such as HIFU. Biopsy negative rate can vary from 75.5% in low-risk patients to 18.7% in very high risk patients following single session HIFU [9]. Currently indications to use HIFU include T1-T2, Nx-N0, M0 patients who are not fit to surgery, for example, age > 70, life expectancy < 10 years, or presence of comorbidities [11].

## 5. Conclusion

Though salvage laparoscopic prostatectomy has been associated with higher morbidity than primary surgery in men who have previously undergone HIFU treatment, it was shown that the utilization of RALP performed by an experienced surgeon can be a safe and an effective treatment choice for patients with recurrent prostate cancer.

## Additional Points

*Key Messages.* (i) Oncologic outcome of prostate cancer patients following HIFU is yet to be studied. (ii) Sexual function and urinary control is maintained at high rates after robotic assisted laparoscopic prostatectomy at experienced centers. (iii) Robotic assisted laparoscopic prostatectomy remains the gold standard of treatment in prostate cancer.
